# Pathology

**Published:** 1878-12

**Authors:** 


### Pathology.

Renal Calculus largely composed of Indigo. (*Br. Med. Jour.*, July 27, 1878.)

In a clinical lecture at St. Thomas' Hospital, Dr. Ord exhibited two calculi, removed from the kidneys of a patient dying of malignant disease, one of which consisted very largely of indigo. That there was here no mistake, the application of several tests as well as spectrum analysis conclusively proved. More definitely, the calculus consisted of a matrix of calcic and magnesic phosphates, with a few remains of blood clot, the matrix being everywhere penetrated by indigo-blue and a little indigo red, while indigo blue was deposited in large proportion as incrustation.

With reference to the ætiology of this rare formation, the following is summarized from his lecture. Indican and indigo-blue are formed spontaneously in the urine, or by aid of acids and oxygen; hence the test for indican. As Schunk explains the process, it is thus:

$$\underset{\text{Indican.}}{C_{26}H_{31}N\,O_{17}} + 2\,H_2O = \underset{\text{Indigo-blue.}}{C_8H_5N\,O} + \underset{\text{Indiglucin.}}{3\,C_6H_{10}O_6}$$

Indigo-blue has been found in the blood of man and animals. The appearance of indigogenous material in urine may be explained in two ways; it may be due to the ingestion of indigogenous material, or to the breaking down of nitrogenous matter in the body. The use of carbolic acid and creosote is often followed by a blackening of the urine, due to the above. The urine of starving animals, also, often contains it. It may be a derivative of blood pigment, or of albuminous bodies. Long ago Dr. Hassal pointed out the chemical similarity between the coloring matter of urine, indigo and blood pigment. Recent observations give reason to believe that indigo-blue in the urine is connected with the formation and subsequent oxydation of indol ($C_8H_7N$) in the system. Indol is one of the results of the action of pancreatic juice on peptones, and is found in the fæces, which owe much of their odor to it. Its subcutaneous exhibition is followed by appearance of indigo blue in the urine. Pus, also, has often a greenish or bluish color, which is caused by the same material.

Comparing experimental with clinical observations it may be inferred that several conditions will cause excess of indigo in the system ; defective gastric and intestinal chemistry, intestinal obstruction, diarrhœa, suppurations anywhere, but especially in the urinary tract, and morbid processes involving degeneration (amyloid, especially) or destruction of albuminous tissues or blood. In cholera obstruction, and in typhoid the quantity of indigo yielded by the urine is often very large.

It was suggested that, in this case, the indigo was found in the suppurating left kidney in the presence of an alkali, was absorbed and carried by the blood to the right kidney, and then deposited in the presence of uric acid. This was considered plausible. The patient had taken a little creosote, and he was markedly cachectic ; thus the conditions were all favorable.

On Excision of the Primitive Sclerosis of Syphilis. Prof. H. Auspitz. (*Vierteljahresschrift für Derm. u. Syph.*, 1877.)

The author gives the history of 33 cases in which he excised the primitive induration. The operation was never followed by any evil result either local or general. On the contrary, locally, the cicatrix was often seen to present no induration, and when the excision did not prevent the outbreak of secondary accidents, there was always a delay in their appearance and a certain benignity.

In the 33 cases three were null ; two were lost sight of and one had roseola at the time of operation, the object being microscopic examination of the ulcer.

In 20 cases no syphilitic manifestation appeared after excision ; in 10 others secondary lesions appeared, with the benignity of character already mentioned.

In all these observations, taken for the most part from the polyclinic at Vienna, the patients were followed four months, and sometimes eighteen months.

Auspitz concludes thus: "To prevent syphilis it is necessary to excise the initial sclerosis. It is necessary that this sclerosis should be recent, and not have presented other complication than the indolent inguinal adenitis which is always observed; it is necessary also that the operation and the dressings may be made easily."

